# Amiloride as an Alternate Adjuvant Antiproteinuric Agent in Fabry Disease: The Potential Roles of Plasmin and uPAR

**DOI:** 10.1155/2014/854521

**Published:** 2014-05-15

**Authors:** H. Trimarchi, M. Forrester, F. Lombi, V. Pomeranz, M. S. Raña, A. Karl, J. Andrews

**Affiliations:** Nephrology Service, Hospital Británico de Buenos Aires, Perdriel 74, 1280 Buenos Aires, Argentina

## Abstract

Patients with Fabry disease present a higher risk of cardiovascular and kidney morbidity. We present a patient with a past history of biopsy-proven Fabry disease and stage 3 chronic kidney disease. Proteinuria partially dropped from 6.8 g/day to 2.1 g/day despite an aggressive regime which consisted of low-salt diet, agalsidase beta infusions, dual blockade of the renin-angiotensin system, and low-dose maintenance of steroids. As proteinuria is considered a risk marker of cardiovascular disease and of progression of kidney disease, we added amiloride 5 mg/day, a drug with proven effects in podocyte stabilization and proteinuria actions at the distal convoluted tubule. Proteinuria finally decreased to 0.8 g/day. This report highlights the relevance of intervening on proteinuria in a multitarget approach in order to reduce it as much as possible. Due to this pharmacological response, we suggest that although agalsidase beta specific treatment protects the endothelium, the podocyte, and the tubule in Fabry disease and secondary haemodynamic and immunologic pathways are treated with inhibition of the renin-angiotensin system and steroids, amiloride may act as a complementary tool in podocyte stabilization and in proteinuria effects at the distal tubule.

## 1. Introduction 

Fabry disease is a hereditary disease with systemic involvement, mainly affecting the cardiovascular, renal, and neurologic systems. Despite specific replacement therapy, renal involvement is progressive. Proteinuria, a marker of glomerular injury, continues to be elevated in subjects with Fabry disease. We propose an adjuvant therapy with amiloride to be considered in the treatment of these patients, suggesting a rationale for its use.

## 2. Case Presentation

A kidney biopsy was performed in a 37-year-old man with chronic kidney disease stage 3 and Fabry disease was diagnosed, as previously described [1]. Besides the characteristic and specific features of Fabry disease, the biopsy disclosed remarkable and predominating findings consistent with advanced secondary focal and segmental glomerulosclerosis. Despite a wide pharmacological maintenance therapeutic intervention consisting of agalsidase beta for 24 months (Fabrazyme, Genzyme Corp., Cambridge MA), meprednisone 6 mg/day, valsartan 160 mg/day, and aliskiren 300 mg/day, proteinuria persisted between 2 and 2.8 g/day. Other medications included omeprazole 20 mg/day, aspirin 100 mg/day, and ergocalciferol 16800 IU/weekly. Low-sodium diet compliance was followed with urinary sodium excretion, achieving an average 24-hour sodium concentration of 40 mEq/day. Finally, amiloride 5 mg/day was added to this regime, and proteinuria decreased to 0.9 g/day for the last 6 months of follow-up. Noteworthy, antihypertensive drugs were added in a stepwise manner to avoid hypotensive episodes, which were not referred by the patient. The patient was started initially on valsartan 160 mg, followed by aliskiren 300 mg/day four weeks later. Amiloride 5 mg/day was finally prescribed one year later. No hyperkalemic events have been reported.

## 3. Discussion

Fabry disease is an X-linked genetic disorder of glycosphingolipid catabolism resulting from deficient activity of the lysosomal enzyme *α*-galactosidase A. As a consequence, neutral glycosphingolipids, mainly globotriaosylceramide (GL-3), accumulate in a variety of cells and tissues, leading to a wide clinical spectrum of clinical manifestations [2]. Chronic kidney disease (CKD) is a prominent feature of Fabry disease [2, 3] that accounts for 0.01% of end-stage kidney disease patients enrolled in European and US dialysis registries [4, 5]. However, enzymatic screening studies suggest that the true prevalence for male dialysis patients may be 10- to 100-fold higher [6, 7]. Before the advent of dialysis and transplantation, males commonly died of CKD in the fifth decade of life. In a recent study of 106 male Fabry patients, all those surviving to the age of 56 developed ESRD and no patient survived beyond the age of 60 years [3]. Enzyme replacement therapy with human recombinant *α*-galactosidase A has been available since 2001 [8, 9]. Although family studies and case reports have disclosed some aspects of Fabry nephropathy, the rarity of this disorder has made it difficult to fully appreciate the spectrum of kidney involvement in male and female patients [10].

**Figure 1:**
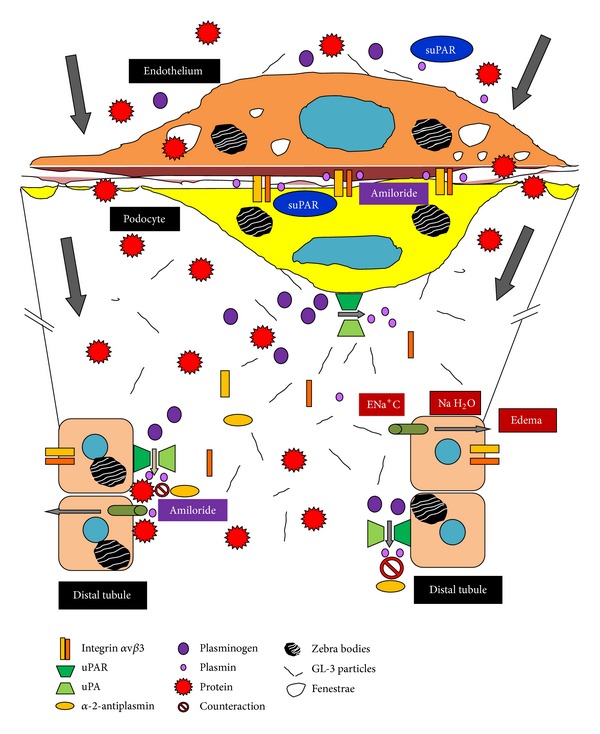
Molecular mechanisms of proteinuria in Fabry disease and amiloride effects.

At the cellular renal biotype level, podocytes, endothelial cells, tubular cells, and mesangial cells are injured and, consequently, the glomerular basement membrane and the interstitium are involved, resulting in proteinuria and eventually in renal failure (Figure 1). As we previously outlined [1], the suggested mechanisms of renal injury in Fabry disease include vascular compromise secondary to deposition of GL-3 within the arterial wall, which should be considered as the* first hit*, with a concomitant decrease in nitric oxide synthesis and a tendency to microthrombotic events, podocyte injury and detachment with secondary glomerulosclerosis, and tubular atrophy and interstitial fibrosis [11]. Albeit a specific treatment for the disease exists, proteinuria frequently persists, particularly as renal disease worsens [10]. Inflammatory and hemodynamic pathways are subsequently triggered, justifying the employment of steroids and drugs that interfere with the local renin-angiotensin system, as angiotensin converting enzyme inhibitors, angiotensin receptor blockers, or aliskiren. This conundrum of inflammatory and hemodynamic factors could be considered as a* second hit* of Fabry disease in the kidney [1].

In our case report, proteinuria significantly decreased from 6.8 g/day to 2 g/day with a multitarget approach. However, this level of proteinuria is still elevated for a subject with CKD. Proteinuria is not only a marker of progression of renal disease, but also a risk factor of cardiovascular disease in the general population [12–15]. Numerous studies have shown that treating patients with diabetic/nondiabetic CKD and proteinuria reduces proteinuria and slows the progression of renal disease and that the greater the proteinuria decrease, the greater the benefit [16–18]. The cardiovascular risk is already elevated in Fabry disease due to the accumulation of GL-3 in the heart [19]. The cardiovascular manifestations of Fabry disease include hypertension, left ventricular hypertrophy, rhythm and conduction abnormalities, increased intima and media thickness, valvular insufficiency, and ischemic heart disease [20–22]. Over time, these cardiac complications can progress to heart failure, myocardial infarction, and life-threatening arrhythmias [23–25]. However, the incidence rate and predictors of cardiovascular events in patients with Fabry disease are not well known. Therefore, it is reasonable to presume that lowering proteinuria could retard the progression of kidney disease and cardiovascular events in Fabry disease by decreasing blood pressure, hyperfiltration, sodium intake, inflammation, and adequate enzyme replacement therapy. Although the nature of the links between proteinuria and vascular disease may partly be due to endothelial dysfunction, persistent low-grade inflammation also plays a role. Indeed, inflammation is associated with both endothelial dysfunction and albuminuria [26, 27]. Moreover, in Munich-Wistar-Fromster rats, glycocalix endothelial damage at the glomerular level is the starting point of secondary distant endothelial derangements that result in cardiovascular structural derangements [28] and could explain the already mentioned clinical outcomes. Therefore, strategies to reduce proteinuria are to be considered as adjuvant therapies. In this regard, steroid treatment could be controversial due mainly to its side effects profile, particularly in secondary cases of focal and segmental glomerulosclerosis. but as detailed in our previous report, meprednisone was used at low doses to modulate inflammatory pathways involved in heavy proteinuria management [1].

**Figure 2:**
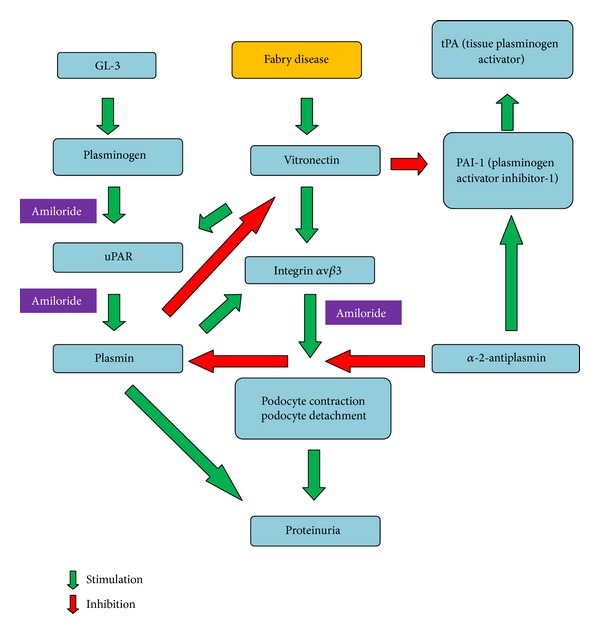
Interactions between globotriaosylceramide (GL-3), uPAR, plasminogen/plasmin, and integrin *α*v*β*3 and the development of proteinuria in Fabry disease, counteracted by amiloride.

Amiloride plays a significant role in reducing podocyte cell motility in vitro and proteinuria in mice [29]. Besides its diuretic properties at the distal tubule blocking the absorption of sodium and water at the ENa^+^C channel, its well-tolerated drug profile, and its low cost, amiloride has been recently shown to inhibit the synthesis of the urokinase receptor, called urokinase plasminogen activator receptor (uPAR), a molecule involved in the *α*v*β*3 integrin activation [29–31] (Figure 2). This integrin (also known as the vitronectin receptor) anchors the podocyte to the glomerular basement membrane; when activated it causes podocyte contraction and eventually contributes to the detachment of the cell from the glomerulus and its appearance in the urine (a phenomenon known as podocyturia), finally causing proteinuria. The reduced uPAR pool caused by amiloride would also translate into a lower suPAR concentration, the soluble circulating version of uPAR but also with glomerular permeability factor properties [32] (Figure 1). Amiloride capacity to inhibit uPAR synthesis and suPAR secretion by T lymphocytes, macrophages, and neutrophils should be of particular interest in Fabry disease, because blocking their activation would inhibit *α*v*β*3 integrin activation and another route of proteinuria [30, 31]. Interestingly enough, Utsumi et al. have reported that the urinary excretion of *α*v*β*3 integrin is elevated in subjects with Fabry disease. Increased expression of the *β*3 component was observed in glomerular epithelial cells and in Bowman's capsular epithelial layer and tubular cells, and the amount of vitronectin (a molecule involved in adhesion and fibrinolysis) was moderately increased in the kidney from Fabry patients. The urinary excretion of the integrin *α*v*β*3 was also increased and its expression was also observed in Fabry kidney tissues (Figures 1 and 2). Therefore, the expression of the integrin *α*v*β*3 may be involved in podocyte contraction and eventual detachment from the glomerular basement membrane and could be another pathophysiological cause of proteinuria, finally contributing to the progression of renal injury in Fabry disease [33].

Furthermore, amiloride may further decrease proteinuria by acting on the distal nephron in ENa^+^C channels, as proteinuria stimulates the activity of these channels by promoting the reabsorption of sodium and water [34]. Tubular plasmin, already high in patients with proteinuria, would act as the mediator in sodium and water reabsorption and amiloride may inhibit its action by blocking uPAR [29, 34–36] (Figure 1). Thus, this would be another additional and relevant nonimmunosuppressive strategy contributing to the fall in proteinuria, if tolerated hemodynamically and no hyperkalemia ensues [32].

The fact that our patient has responded to amiloride gives clinical support to the previously mentioned findings; that is, in Fabry disease integrin *α*v*β*3 and uPAR expression may be elevated and involved in the pathogenesis of proteinuria and eventually in the progression of kidney disease. This interaction could occur in the podocyte and at the distal tubule. To our knowledge, there is no data available relating Fabry disease directly with uPAR or suPAR, although it has been suggested by Reiser [37]. The published evidence about *α*v*β*3 integrin activation (a target of uPAR) in Fabry disease deserves for this tempting hypothesis to be proven.

Moreover, it has been demonstrated that in subjects with Fabry disease, both plasminogen, plasmin and alpha-2-antiplasmin levels are elevated, and its secondary decrease is due to overconsumption of these factors [38] (Figure 1). Briefly, uPA catalyzes the conversion of plasminogen to plasmin. In this setting, endothelial dysfunction results in either low-grade secretion of tissue plasminogen activator (tPA) or enhanced plasminogen activation on the surface of the endothelial cell by urokinase or UPA (urokinase-type plasminogen activator) in subjects with Fabry disease [38]. The latter hypothesis is also attractive because gangliosides play an important role in the binding of plasminogen to the cell surface [39]. Globotriaosylceramide is normally present on the cell surface and is elevated in Fabry disease [33, 40]. Therefore, an increased content of *α*-D-galactosyl-containing sphingolipids in Fabry disease in the cell membrane may cause enhanced activation of plasminogen to plasmin by uPA or urokinase [38] (Figures 1 and 2). Alpha-2-antiplasmin (a plasmin inhibitor that controls plasmin-mediated fibrinolysis), plasminogen, and tPA concentrations in the blood and urine would be useful markers to be measured also in patients with Fabry disease who are without enzyme replacement therapy and follow their levels after treatment. Finally, the role uPAR and suPAR may play in proteinuria as well as the binding and activation of plasminogen to plasmin on the surface of renal endothelial, podocyte, and tubular cells of patients with Fabry disease is another subject to be studied, as uPAR/suPAR could be involved in the pathogenesis of proteinuria and amiloride could be a potential adjunctive tool to reduce it, modulating inflammation and thrombogenic mechanisms in Fabry disease.
